# We Need to Stay Flexible: Making a Case for Fiberoptic Intubation Skills

**DOI:** 10.7759/cureus.97551

**Published:** 2025-11-23

**Authors:** Fahd Sahi, Haroon Mather, Peter Murchan, Marcella Lanzinger

**Affiliations:** 1 Anesthesiology and Critical Care, Tipperary University Hospital, Clonmel, IRL; 2 General Surgery, South Tipperary General Hospital, Clonmel, IRL

**Keywords:** airway management, anesthesia, awake fiberoptic intubation, difficult airway, head and neck cancer, skill retention, video laryngoscopy

## Abstract

Advances in video laryngoscopy (VL) have revolutionized airway management with higher successful first-attempt intubation rates as compared to direct laryngoscopy. However, in select patients with complex airway anatomy, particularly those with head and neck cancer, awake fiberoptic intubation (AFOI) remains the gold standard. The growing preference for VL risks marginalizing AFOI, potentially disadvantaging patients who cannot be safely intubated otherwise. We present the case of a 61-year-old male with a history of advanced throat cancer and prior extensive head and neck surgery, who presented with rectal bleeding and was diagnosed with sigmoid cancer requiring urgent colectomy. Preoperative airway assessment revealed multiple predictors of a difficult airway, including recurrent pharyngeal tumor extending into the larynx, subglottic stenosis, limited neck mobility, and distorted anatomy. In a prospective multidisciplinary team approach, AFOI was successfully performed using flexible fiberoptic bronchoscopy under anxiolysis and topical anesthesia, with emergency front-of-neck access on standby. The airway was secured uneventfully as planned. The patient was extubated at the end of surgery without complication and discharged from the ICU on postoperative day 2. This case underscores the critical importance of preserving fiberoptic intubation skills for managing difficult airways. VL is not always feasible in anatomically altered or surgically reconstructed airways. Despite its decreasing frequency in clinical use, fiberoptic intubation remains a valuable, potentially lifesaving, technique. Simulation and team-based approaches are essential for skill retention and preparedness. In an era dominated by VL, AFOI must remain a core competency for anesthesiologists managing complex airways. Continued training, simulation, and clinical application are vital to prevent the extinction of this critical skill.

## Introduction

In the face of emerging technology, older skillsets sometimes fade away. Direct versus video laryngoscopy (VL) trials have suggested differing outcomes on the advantages of VL [1,2], but a recent meta-analysis suggests that VL results in a higher rate of first attempt intubation success [3]. In the management of difficult airways, awake fiberoptic intubation is considered the Gold standard compared to awake video laryngoscopic intubation [4]. However, with recent advances, VL may be considered the first choice in difficult airway management [5]. In the face of the emerging and increasing use of VL, awake fiberoptic intubation, which is already a rarity, could become endangered altogether. This issue of changing practice for the greater good leaves a smaller number of patients potentially disadvantaged and puts patients at risk for whom airway management with awake fiber-optic intubation is the only choice [6].

## Case presentation

A 61-year-old male presented to the emergency department with complaints of rectal bleeding. He was assessed by the surgical team and subsequently admitted with a Hemoglobin of 6.8 g/dl (Table 1). The patient was stabilized, and upon further investigation, he was diagnosed with sigmoid cancer and scheduled for urgent sigmoid colectomy due to ongoing bleeding not amenable to endoscopic intervention.

**Table 1:** Laboratory investigations showing low hemoglobin and hematocrit (presented in bold font) WBC: white blood cell, RBC: red blood cell, HB: hemoglobin, HCT: hematocrit, MCV: mean corpuscular volume, MCH: mean corpuscular hemoglobin, MCHC: mean corpuscular hemoglobin concentration

Test	Result	Unit	Normal range
WBC	4.6	x10^9^/L	4-10
RBC	2.42	x10^12^/L	4.5-5.5
HB	6.8	g/dl	13-17
HCT	0.21	L/L	0.40-0.50
MCV	84.7	fl	83-101
MCH	28.1	pg	27-32
MCHC	33.2	g/dl	31.5-36
Platelets	155	x10^9^/L	150-400
Neutrophils	3.40	x10^9^/L	2-7
Lymphocytes	0.64	x10^9^/L	1-3
Monocytes	0.42	x10^9^/L	0.2-1.0
Eosinophils	0.06	x10^9^/L	0.02-0.5
Basophils	0.06	x10^9^/L	0.02-0.1

**Figure 1:**
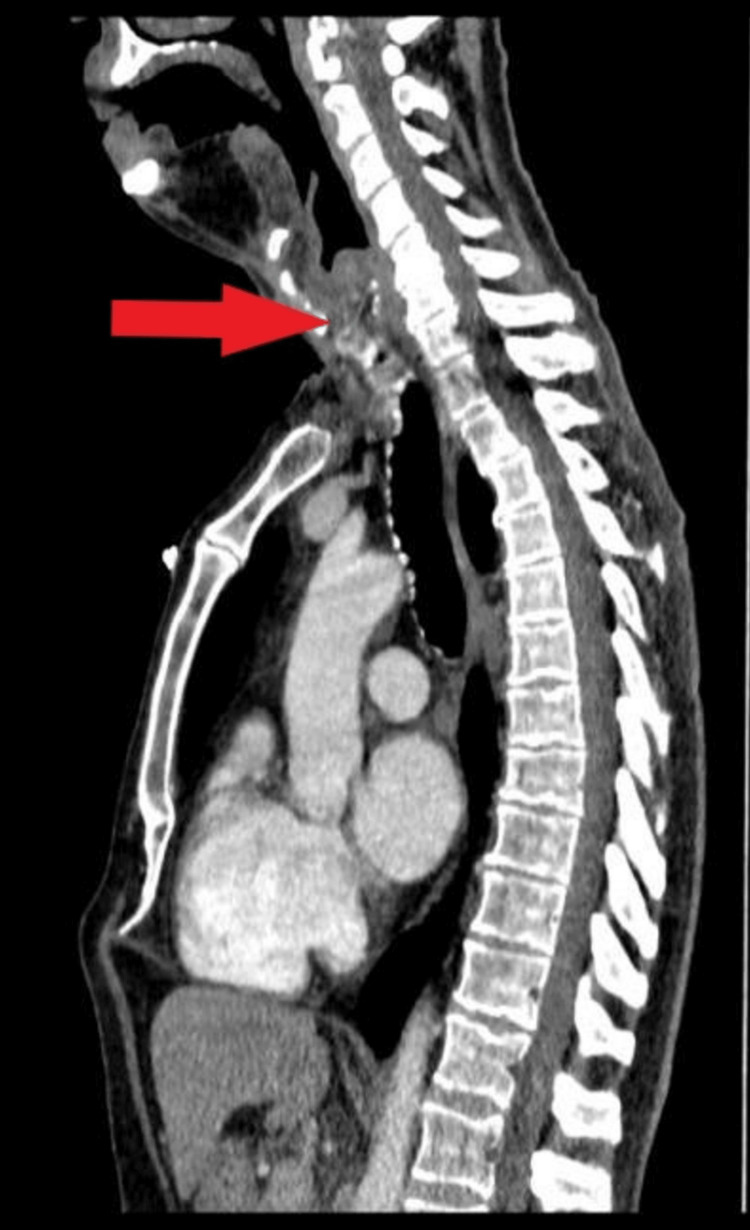
CT scan: red arrow pointing towards subglottic stenosis.

**Figure 2:**
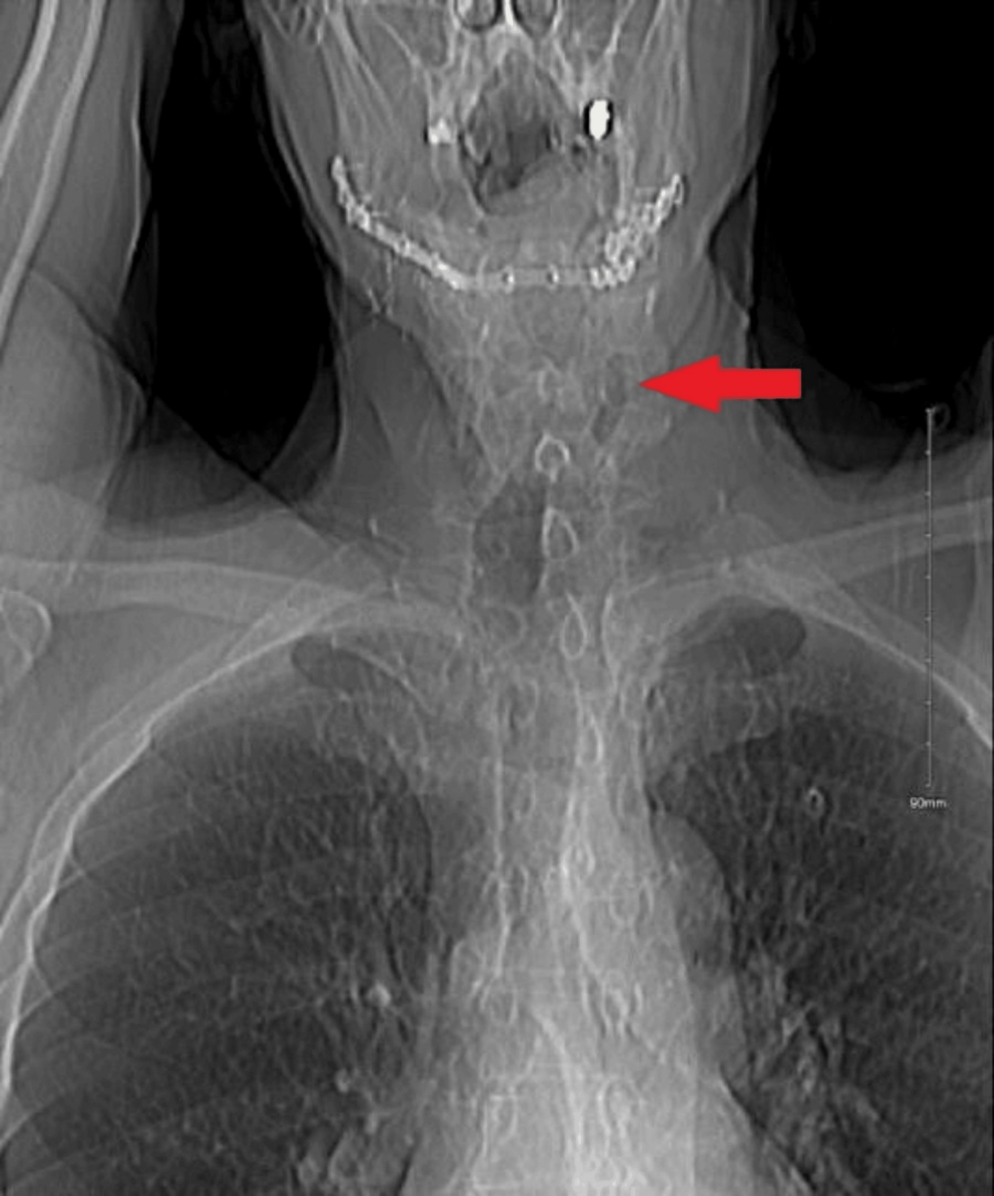
X-ray chest: red arrow pointing to left-sided deviation of the trachea.

The patient was discussed preoperatively by a multidisciplinary team. On preoperative assessment by the anesthesiologist, the patient was awake, conscious, and oriented. He had a documented history of throat cancer diagnosed two years earlier, treated with radiotherapy and surgical resection with reconstruction of the oropharynx. Surgery included left-sided hemi-glossectomy, mandibulectomy, bilateral neck dissection, and oropharyngeal reconstruction with soft tissue flap and fibular graft to mandible. Current localized infection at the anterior mandibular reconstruction site. Vital signs were BMI of 24, heart rate 47, blood pressure 130/70 mmHg, and SpO_2_ 98%. Poor airway protection with silent airway penetration and chronic aspiration, dyssynchronous limited swallowing, and gastroesophageal reflux symptoms were documented. There was no oral input since surgery. He also denied shortness of breath or breathing limitations. On airway assessment, he had a moderately limited range of motion of the neck, with a significantly indurated right side of the neck, good mouth opening, and a Malampati view of grade 4 [7]. There were very narrow nasal passages bilaterally. On CT and X-ray, there was documented new recurrence of tumor extending into the larynx with subglottic stenosis, side-to-side deviation, and narrowing of the subglottic trachea on the left (Figures 1, 2).

The anesthesiology team developed a comprehensive plan for securing the airway. All team members in the theatre were informed and briefed prior to the surgery. Equipment and necessary anesthetic, surgical, and technical staff were identified and resourced. The patient was informed of the planned procedure, and his consent and cooperation were secured. On the day of surgery, the planned procedure was again reviewed with the multidisciplinary team and the patient, and individual responsibilities were assigned. Awake oral fiberoptic intubation was planned, with a separate anesthesiologist for anxiolysis and sedation management, as well as a surgical team and equipment on immediate standby for emergency front-of-neck access via cricothyroidotomy or lower tracheostomy.

Standard monitors applied prior to induction. High-flow nasal cannula oxygenation at 100% FiO_2_ (Optiflow^TM^) was used to pre-oxygenate the patient and continued throughout the procedure until the airway was secured. Topical lidocaine 4% was applied to the oropharynx. Fentanyl 100 ug/ml and propofol 100 mg were administered intravenously in increments for a goal RASS -1. Explorative oral fiberoptic investigation of deep oropharyngeal anatomy, followed by brief glottic and subglottic exploration under additional topical anesthesia with 5 ml lidocaine 2%. The fiberoptic probe was advanced successfully without causing tissue damage or bleeding despite a tortuous advancement within the airway due to the altered anatomy of the pharynx. The exploration confirmed the viability of our approach and a patent trachea beyond the glottis. A repeat definitive fiberoptic intubation of the airway was successfully performed, and an endotracheal tube size 6.0 was positioned in the trachea. The patient was subsequently anesthetized with propofol and paralyzed with Rocuronium for Sevoflurane maintenance anesthesia. Invasive monitoring was placed uneventfully under ultrasound guidance with a central venous catheter in the left internal jugular vein and an arterial line in the right radial artery. Oropharyngeal temperature monitoring and depth of anesthesia were monitored with Bispectral index (BIS^TM^). All observations remained satisfactory and within normal limits. At the end of surgery, we confirmed an air leak around the endotracheal tube at 12 cmH_2_O. Quantitative neuromuscular monitoring confirmed complete reversal of block after 200 mg of Sugammadex. The multidisciplinary team was again in immediate standby for potential fiberoptic reintubation or emergency front-of-neck access. The patient was converted to spontaneous ventilation and allowed to emerge from anesthesia. Fully awake and breathing adequately, the patient was successfully extubated onto high-flow nasal cannula oxygen. After a period of observation, he was transferred to the intensive care unit and discharged to the ward on postoperative day 2 without further complication.

At the postoperative anesthetic visit, the patient was very satisfied with the anesthetic care, the inclusive approach to communication, and the executed airway management. He denied any discomfort or procedure-related distress. On further follow-up after discharge from hospital, he confirmed his satisfaction with the care he received.

## Discussion

VL requires space within the oral cavity to maneuver. This can be limited by a smaller mouth opening and in cases of anatomic abnormalities within the oral cavity [6]. Pressure points under the blade can bleed, especially where there is tumor growth [6]. There is little to no subglottic assessment. Our case highlights the significance of flexible fiberoptic skills in managing difficult airways. The patient had tumor progression with obstructed pharyngeal passage and significant bleeding potential. The literature shows that there is a significant decline in the use of fiberoptic intubation [8]. The ease of success with VL for difficult airways is accelerating the decline of fiberoptic intubation and maintenance of skill. Fiberoptic intubation skills after the initial learning phase require regular use to maintain. In the absence of clinical situations, simulation on mannequins might be useful [9,10]. Hereby, mannequins with difficult airways and airway abnormalities should be utilized [9,10]. However, studies appear to suggest that simple simulation training may not be sufficient to improve this skill. The exact reasons are still to be elucidated, but may also be due to the heightened stress of dealing with an alive patient, possibly under pressure. A predictive multidisciplinary team approach and modelling thereof in simulation scenarios may prove beneficial. A team that is trained in the progression of actions will have better communication and preparedness. This, in turn, will avoid delays in patient care when these actions become necessary. In the future, it is envisaged that 3D and AI-supported imaging with robotic techniques may replace fiberoptic intubation [9,10]. Our recommendations, for the present, are to utilize the best current skill maintenance options available at your institution, attend simulation training where possible, and develop a team approach to awake fiberoptic intubation.

## Conclusions

This case highlights the continued relevance and necessity of AFOI in managing complex and anatomically challenging airways, particularly in patients with prior head and neck surgeries or tumor recurrence. While VL has become the preferred technique in many settings due to its ease of use and high first-pass success rates, it is not universally applicable. In cases where oral access is limited or laryngo-pharyngeal anatomy is significantly distorted, VL may fail or even cause harm.

The gradual decline in fiberoptic intubation practice poses a significant risk to patients who rely on it as their only safe airway option. Maintaining proficiency in AFOI through regular clinical use, simulation, and team-based training is essential. Institutions and training programs must prioritize the preservation of this critical skill to ensure safe and effective airway management for all patients, regardless of anatomical complexity.
